# Study protocol: using a mobile phone-based application to increase awareness and uptake of sexual and reproductive health services among the youth in Uganda. A randomized controlled trial

**DOI:** 10.1186/s12978-018-0642-0

**Published:** 2018-12-22

**Authors:** Elly Nuwamanya, Afra Nuwasiima, Janet U. Babigumira, Francis T. Asiimwe, Solomon J. Lubinga, Joseph B. Babigumira

**Affiliations:** 1 Global Health Economics Ltd, P.O Box 27011, Kampala, Uganda; 2 Global Medicines Program, Department of Global Health, University of Washington, P.O. Box 357630, Seattle, WA 98195 USA

**Keywords:** Mobile phone application, Sexual and reproductive health, Youth, In-app advertising, Randomized controlled trial, Economic evaluation

## Abstract

**Background:**

Several cost-effective programs are being implemented around the world that use mobile technology to improve Sexual and Reproductive Health (SRH) uptake and awareness among youth. Mobile phone applications are a viable and effective means of increasing access to SRH services and tools in low and middle-income countries. This paper presents a protocol for a pilot study of a novel program, a mobile phone-based sexual and reproductive health services awareness and delivery application with the objective of increasing the demand for SRH services amongst the youth in Uganda.

**Methods:**

The study employs rigorous evaluation methods to ascertain the impact of the mobile application. We propose a randomized control trial study to determine the causal effect of the mobile phone app in creating awareness and increasing uptake of sexual and reproductive health services in Uganda. The main outcome of the impact evaluation is the percentage change in the SRH services and tools uptake, SRH knowledge and sexual behavior. We will also conduct a model-based incremental cost-effectiveness analysis (CEA) and budget impact analysis (BIA). The main outcomes of the economic evaluation will be the average cost per app user, cost per app service and tool provided. We will also test the in-app advertising model as a way to generate revenue to sustain the program subsidies and related costs.

**Discussion:**

The study seeks to establish the proof of concept of using a mobile application to increase create awareness and increase uptake of SRH tools and services among youth in Uganda. The study results will lead to the development of a demand-driven, culturally-relevant, and easy-to-use mobile app to enhance the uptake of SRH services among the youth in Uganda and globally.

**Trial registration:**

MUREC1/7 No. 07/05–18. Registered 29th June 2018.

## Plain English summary

We purpose to develop a mobile phone application (app) to increase awareness and uptake of sexual and reproductive health (SRH) services among youth aged 18 to 30 years in Uganda. This app is intended to link users to health facilities where they can access SRH goods and services. It will also reduce on the challenge of poor access to SRH services due to lack of privacy and confidentiality: the app allows users to make orders online and goods are delivered to pick up points of individuals’ choice. We will partner with an app developer to develop the app. We will then pilot this study among students of Kyambogo university, and participants who will download the app will be followed up at 1, 3 and 6 months. We will collect data on app usage from the server, and perform analyses to determine technical performance, impact (increase in SRH demand and usage), acceptability, usability, and cost effectiveness. The prototype app includes an advertising interface to enable corporations and other business entities to collaborate as potential advertisers and to generate revenue for sustainability. If successful, we propose to extend the program to all the youth in the country who own a smart phone but, also, include other services.

### Background

Across the globe, youth account for 17.6% of the population size, and in low and middle-income countries (LMICs), while 90% are aged between 10 and 24 years [1]. During adolescence, many young women are at high risk of teen pregnancies, unsafe abortions and as a consequence, maternal and child mortality. Across the globe, over 16 million girls aged 15 to 19 give birth annually [2, 3] and over seven million girls aged 10 to 19 get unintended pregnancies for reasons such as poor access to education and contraception services [2, 4, 5]. These pregnancies lead to over three million unsafe abortions accounting for the largest cause of mortality among women aged 15 to 19 [2]. Of the available sexual and reproductive health (SRH) interventions, the use of modern contraception alone has the potential to prevent approximately 30% of maternal and 10% of child deaths [6]. In addition to preventing the adverse health effects of unintended pregnancies, contraceptive use contributes to the realization of Sustainable Development Goals (SDGs) by limiting the number of unplanned births and child deaths, and increasing the resource envelope that families spend on other necessities using money saved by having planned pregnancies [7].

In Uganda, youth face many SRH risks such as the unmet need for contraception (30%), which leads to unplanned pregnancies (43%) that result in unsafe abortions (30%) and sexually transmitted infections (17%) [8], [9]. The majority of the youth in the country also experience unemployment (62%), engagement in risky sexual behavior (56%), possession of limited knowledge of SRH services (39%) and limited access to SRH services (22%) [10–13].

Awareness and uptake of SRH services remains sub-optimal in LMICs [2, 12, 14], and challenges to the provision of SRH services include lack of privacy and confidentiality, knowledge gaps, lack of finances, cultural and social stigma, biased service providers towards the youth, and inconvenience in accessing SRH services despite their availability [15–18]. Although there have been improvements in creating a youth-attractive environment for SRH services and access to tools, more work is needed [19].

The question researchers and policymakers should be asking is, what works for the youth? Earlier reviews suggest that SRH interventions targeting youth should be accessible, equitable, appropriate and effective [17]. In our understanding, and in the context of Uganda, the challenges to reaching the current generation of poor and young adolescents are mostly related to privacy, confidentiality, and limited SRH knowledge [11].

What strategies are currently in place to improve access to SRH services and tools? A largely successful strategy involved SRH public media campaigns (e.g., through radios, billboards, local skits, television campaigns), while sensitizing the youth [20–22]. Leveraging the high penetration of mobile technology would be a great deal since 27% of phone users are aged under 30 [22, 23], and in Uganda, it’s estimated that 37% of the country’s population has access to the internet [24]. This serves as a potential solution through the provision of high-quality, non-judgmental and non-stigmatizing SRH services.

Is there a role for mobile phone applications to increase SRH access and uptake? It certainly seems smart and possible to leverage the high mobile phone coverage rates to increase SRH services uptake. The literature is replete with evidence that *text messaging interventions* may induce both short and long-term behavioral change, and improve outcomes related to smoking cessation [25, 26], physical activity and obesity [13–19], diabetes, asthma self-management, adherence to hypertension medication and SRH [27–34]. This is because many people can access text messages despite having different types of phones.

Several programs are being implemented around the world that integrate mobile technology to improve SRH uptake among youth, and deliver SRH information, and have been proven to be cost-effective in Kenya and Tanzania [32, 35]. Confidentiality and provision of information that is easy to understand increased demand for family planning information using mobile phones [16, 35]. While several mobile SRH applications exist, most lack sufficient functionality, usability, and effectiveness, measured as long-term, sustainable behavioral change [17, 30, 36].

In a review of the literature, we found some mobile phone apps for SRH [30], a competition and process to develop an SRH application [27] and other projects to develop SRH apps for at-risk youth in LMICs [30]. Despite the high level of SRH information consumption via mobile phone applications, evidence of successful use of SRH mobile phone applications in Uganda is limited, and applications that can increase uptake of SRH services and tools are non-existent.

In this study, we propose to develop a mobile phone-based sexual and reproductive health services awareness and delivery application with the objective of increasing the demand for SRH services amongst the youth in Uganda. Our proposed innovation bridges the privacy and confidentiality gap by allowing users to make orders online using the application and have them completed and delivered to their preferred pick-up points. Using the application, users will also be able to schedule a visit to any of the partner clinics. The proposed innovation will reduce both the knowledge and financial barriers to access of SRH information and services by providing comprehensive SRH information and subsidizing SRH services. In the long run, the SRH App will be self-sustaining through revenues from in-app advertisements.

The concept of using mobile apps to link users to services, make orders online, and have orders delivered is itself not novel. Mobile apps like Uber [37] for car services, SafeBoda [38] for boda-boda services, and Jumia [39] for shopping and food deliveries have attracted huge demand from the public. We propose to leverage this service delivery model but with a key difference: instead of users paying for services, we propose to test a model of revenue generation that us dependent on in-app advertising. Some marketers argue that in-app advertising offers users a good experience, especially if highly tolerable and likable ads are designed [39].

In this protocol, we describe a pilot study to develop and evaluate a mobile phone application (app) aimed at increasing uptake of SRH services among the youth in Uganda. We anticipate that this study will lead to the development of a demand-driven, culturally-relevant, and easy-to-use mobile app to enhance the uptake of SRH services among the youth in Uganda and beyond. In addition to reducing the financial and knowledge gap in SRH services, the app that is planned for development and pilot testing in this study is unique in three ways: 1) it will be developed in a collective process that involves local stakeholders and the target population in the design process to ensure high acceptability and user satisfaction, 2) it bridges the gap of privacy and confidentiality by allowing users to anonymously make orders online, and have them delivered at their preferred pick up points and also allows users to choose a visit to a preferred clinic, and 3) it will have an advertisers interface to generate revenue to sustain SRH service subsidies. The objectives of this study are to 1) develop a *mobile phone-based app to increase awareness and uptake of SRH services among the youth aged 18 to 30 years in Uganda, 2)* assess the effectiveness of *the app* on SRH uptake and awareness among the youth *aged 18 to 30 years in Uganda*, 3) determine the acceptability and usability of the app among the youth *aged 18 to 30 years in Uganda, 4) assess the cost-effectiveness and budget impact of the app services delivery model from the perspective of the Ugandan government and taxpayer, and 5)* pilot test in-app advertising as a way of generating revenues to sustain the SRH service subsidies associated with the app.

## Methods

### Study design

**Fig. 1:**
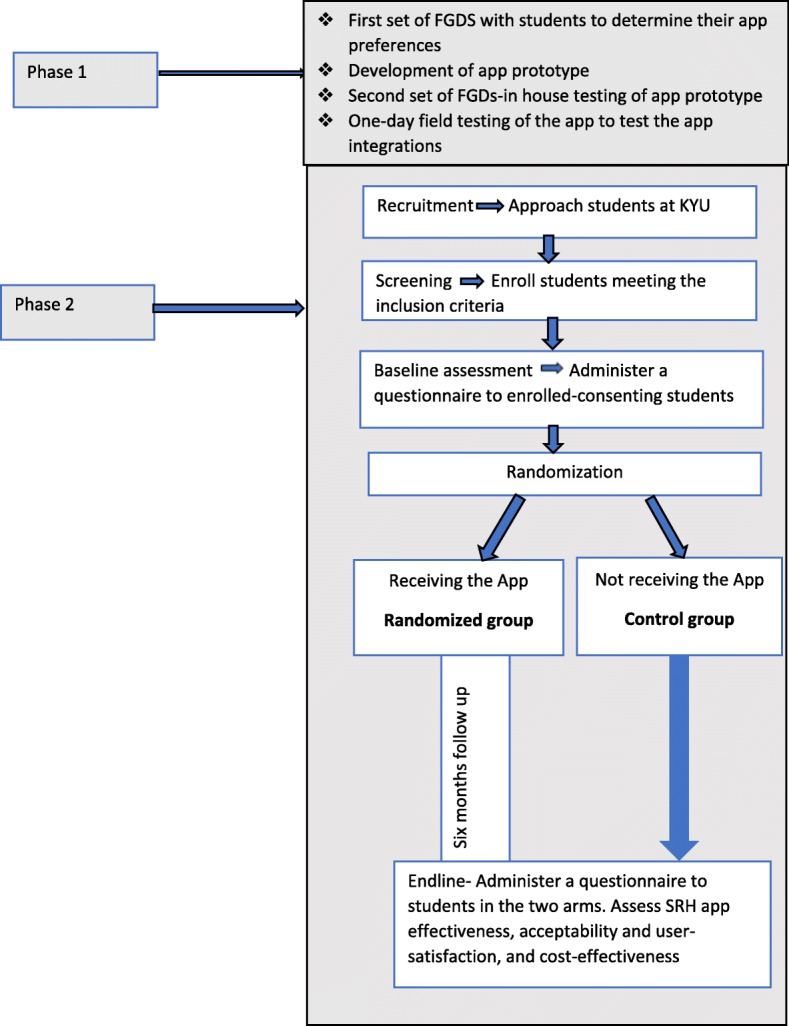
Flow diagram of the SRH App study design. Shows steps that will be taken to design and test the app for impact

This is a pilot project that will be implemented in two phases. In the first phase, we will use a stakeholder-driven approach to develop the SRH mobile app. This will involve Focus Group Discussions (FGDs) to understand app preferences, followed by iterative app design by the app developer, in-house testing through subsequent FGDs and a one-day field test of how the app runs in its current status. The second phase is a prospective impact evaluation that will involve three complementary sub-studies: 1) a randomized controlled trial to evaluate the effect of the app on SRH services awareness and uptake, 2) a cross-sectional study to assess the acceptability, user satisfaction, and the use of in-app advertising for revenue generation, and 3) a cost-effectiveness and budget impact analysis. Details on the study design are presented in Fig. 1.

### Setting

The primary setting of this pilot project is Kyambogo University (KYU) in Kampala, the second largest university in Uganda. The campus is located 8 KM from Kampala city center along the Kampala-Jinja highway on Kyambogo hill. Kyambogo University has a population of over 25,000 students with a near-100% cell phone penetration and it is projected that more than 50% of students own internet-enabled phones, giving an estimated 12,500 eligible participants. The app will be tested among students aged 18 to 30 years residing in the university residency halls and hostels.

### Intervention

#### Proposed SRH app description

**Fig. 2:**
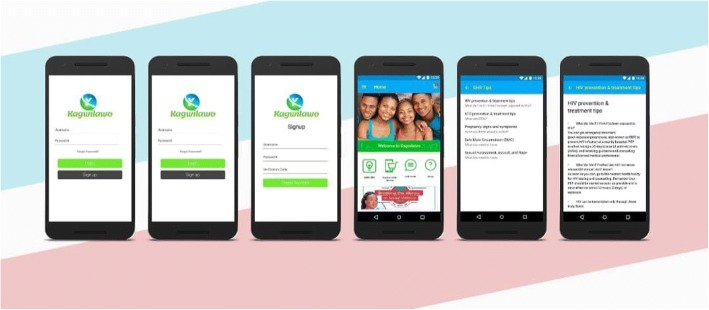
Gives a snapshot of some of the modules as they appear in the App. The word "Kagwilawo" literally meaning "Instant" is the name of the App

We propose to design a user-friendly SRH services awareness and delivery mobile application. The app will create an SRH tool delivery platform where different SRH tools such as condoms, contraceptive and emergency pills, home testing kits, etc. can be ordered and delivered to the user at their preferred pick-up point at a subsidized cost. The app will also link users to their preferred clinics, chosen from a pool of nearby clinics providing SRH services like HIV/STI/pregnancy testing and counseling, and contraception placement. During the study, we will cover their cost of transport to and from the selected clinic. The app will also include: (1) a menstrual cycle tracker for women to track ovulation and menstruation with an aim of understanding when they are at highest risk of becoming pregnant, (2) an interface with SRH tips and tools, and (3) a chat box where users can ask any questions anonymously and receive responses. We also propose to build an advertisement interface and test its use as a way of generating revenues for sustainability. Details on the app modules are presented in Table 1 and Fig. 2.

**Table 1:** Proposed modules of the sexual and reproductive health app

Module	Description of the module	
1. User module	This will be the end user that will most frequently use the application to request for services or toolkits. These will be provided through theapplication. All features as described below will fall under this module.	
	a) Services and products module	This sub-module will contain a list of services and products the app will provide. It will also enable the user made orders for the over the counter products or book/ schedule appointments for the services
	b) SRH tips	This sub-module will contain preventive and control information on sexual and reproductive health care. It will display information in text, figure and video formats.
	c) Period tracker	This sub-module will contain an easy to use period tracking calendar. Females participants will easily track their periods using this tool
	d) Chat box	This sub-module will enable the users of the app chat with the trained counsellors and nurses at the health facilities.
	e) FAQs	This sub-module will contain frequently asked questions. It will be routinely updated.
2. Administrator Module	This module will contain functions that will be used by the GHE and APP administrators to manage health centers make payments, export data/reports, and the all other admin features that will be used at the GHE and APP admin levels. This will be accessed on web portal by the administrators to carry out admin functions.	
3. Payment Module	This module will integrate with Beyonic payments system. This will enable users pay for the goods and services, GHE make payments to Health providers and SafeBoda for deliveries made. This module will be managed via the web by the GHE administrator	
4. Delivery Module	This module will track all the orders made by the users and will collect user’s data on preferred pick-up points. This module will be managed via the app by the Health facility administrators who will package and send order tools to the users using SafeBoda. It will have a tracking task where users and health facility admins cankeep track of their items.	
5. Security, authentication and related Modules	These modules will enable the App to be password protected and authenticated.

The app will be integrated with three existing service providers: (1) partner health facilities for SRH service provision, (2) SafeBoda for transportation, and (3) Beyonic Uganda Ltd. for managing the payments system.

#### Subsidies

We propose to provide a subsidy for every tool or service ordered or accessed using our SRH app. We will pay 50% discount of every SRH service cost at the clinic and the client will pay 50%. We propose to provide a 50% discount on the delivery cost incurred by transporting the tool from the clinic to the preferred pick-up point or picking the user from their preferred location to the clinic. We anticipate that revenues from the in-app advertisements will sustain the subsidies after the pilot phase.

### Control group/comparator

The study control group will receive no intervention but will however be followed at one and six months respectively.

### Study participants

This study will be conducted among the male and female students of Kyambogo University in Uganda. The participants will be included or excluded from the study based on the following criteria;

- *Inclusion criteria*

- Kyambogo University students between the ages of 18–30 years
- Self-reported sexually activity in the past six months
- Left with more than one academic year to completion
- Willing and able to provide written informed consent to participate in the project
- Access to an internet-enabled android smart-phone device

b) *Exclusion criteria*

- Not sexually active i.e. never had sex and no self-reported sexual activity in the past six months
- Refusal to provide informed consent
- Final year at the university
- Not in possession of an internet enabled android smart-phone device

### Study phases

#### Phase 1—App development

- *Study design*

We will employ qualitative methods involving Focus Group Discussions (FGDs) to guide app development. The key stakeholders in this phase of the project include the students (intended target of the application) and the Behavioral Change Communication (BCC) group at the Community Health Department within the Division of Reproductive Health in the Ministry of Health. We have enlisted the BCC group to support our team in development of the SRH tips in the application. They will be involved in design and conduct of the FGDs and in-house testing of the app. The app will be developed in collaboration with an experienced local app developer, and students at Kyambogo University.

b. *Focus Group Discussions*

We propose to conduct two sets of Focus Group Discussions (FGDs) each lasting 45 to 60 min with students from Kyambogo University. In the first set, we propose a maximum of 6 FGDs of 8–10 students each—3 male only and 3 female only groups. The second set consists of 2 FGDs, of 8–10 students each stratified by sex (1-male only and 1-female only) and will be used for in-house testing of the app. We have proposed to use FGDs separated by sex to encourage freedom of expression. However, to encourage diversity of opinion we will attempt to capture a broad age range (potentially defined by the number of years of study at the university) and whether or not they are residents and non-residents of on-campus student halls. The first set of FGDs will be used to: 1) understand students’ perspectives and experiences of what drives risky behavior, and 2) understand the SRH app preferences that students are likely to respond to. The interviews will be semi-structured, comprising of open-ended questions guided by an interviewer. We shall make every attempt to encourage the flow of conversation among the students, only using probing questions where we feel we need more information. We anticipate saturation to occur after 6 FGDs. The second set of FGDs will be used to preview the app and provide feedback to the development team for improvement. We anticipate that after the 2 discussions, the final iteration of the app will be ready for deployment. All FGDs will be recorded with two digital voice recorders and supplemented by notes taken by two interviewers. Focus group data will be transcribed by a professional transcriber. Interviews will be anonymized and transcribed and then entered into Atlas Ti scientific software as primary documents from which codes and patterns will be generated. New themes will also be generated from open coding, and key themes will be identified to beef up the quantitative analysis. The research team will synthesize the data to generate content and design for the app.

c. *App design*

Data from the FGDs will be used to guide the design of the SRH mobile app on an Android platform. We propose to contract with a locally established app developer to design the app. The app developer will participate in both the first and in-house app testing FGDs and in the one-day field event. After the final version of the app has been developed, it will be deployed on the Google Play store where study participants will be able to download and use it. Although we propose to design an app that is driven by the target audience’s preferences, we have enlisted the participation of a Behavioral Change Communications (BCC) expert from the Ministry of Health and an IT expert from the National Information Technology Authority (NITA) who will ensure that the app is consistent with recommended standards for Uganda and conforms to all local regulatory requirements. All versions of the app will be submitted to the Mbarara University of Science and Technology (MUST) Institutional Review Board (IRB) before in-house testing and prior to the pilot testing in phase 2 of the study and, later, to the Uganda National Council for Science and Technology (UNCST) for clearance. After the iterations of app design are complete, we will conduct a one-day field test of the app to test the app’s integration with the partner health facilities, SafeBoda, and the Beyonic payments system. We will also test the responsiveness of the clinics in preparing the orders and the average time it takes for the SafeBoda to connect to the clinic and deliver the package to the client. Based on the outcomes from this activity, recommendations will be made to improve the process.

#### Phase 2—App effectiveness, acceptability, user-satisfaction, cost-effectiveness and in-app advertising

- App effectiveness, acceptability, user-satisfaction

### Study design

We will employ a randomized controlled trial design. Consenting participants will be randomized to download the app or to a control group. The app will not be transferable from one device to another, except through download from Google play store.

### Study treatment

The study treatment is randomization to receive the app. We anticipate that the app will be available for download from Google play store and will be password protected. To ensure that the treatment is not contaminated, we propose that the app developer use a unique one-time password, only useable once by a person (telephone number/ username) that have been randomized to receive the app.

### Pre-study mobilization, sensitization, and training

We will seek administrative clearance and operational guidance from the Reproductive Health Division within the Ministry of Health and the Kampala Capital City Authority to implement the project at Kyambogo University, Kampala. We shall work together with the University administration and student leadership to sensitize participants and encourage participation at recruitment.

This project will be implemented by GHE Consulting researchers, who will undergo rigorous training in research methods, research ethics, and Sexual and Reproductive Health (SRH). We will enroll four clinics and pharmacies that provide high-quality SRH services and are located in the neighborhood of KYU. The enrolled clinics and pharmacies will be integrated with *the SRH app* and will be able to receive and process orders from the clients. We will employ trained personnel at every clinic to manage the orders, pack, and deliver products ordered. The team will also manage the appointments for clients requesting for services at the clinics via the SRH app. We will work with SafeBoda to deliver the products to the preferred pick up points as shall be suggested by the client. We will also integrate with Beyonic Uganda Ltd. to manage the clinic payments and receiving of payments for services from the clients via mobile money.

### Sample size calculation

The primary outcomes of the app effectiveness study are the changes in the prevalence of modern contraceptive use, STI testing, STI treatment, and SRH awareness. We propose to estimate the study’s sample size based on the prevalence of modern contraceptive use among the youth aged 18 to 30 years. According to the 2016 Uganda Demographic and Health Survey, the prevalence of modern contraception among the youth aged 18 to 30 years in Uganda is 30.2%. The study utilizes the sample size formula for Z test for two sample proportions used for behavioral studies to compute sample size estimates.

Sample size formula;

$$ n=d\left(\frac{{\left({Z}_{1-\alpha /2}\sqrt{2p\left(1-p\right)}+{Z}_{1-\beta}\sqrt{\left({p}_1\left(1-{p}_1\right)+{p}_2\left(1-{p}_2\right)\right)}\right)}^2}{{\left({p}_2-{p}_1\right)}^2}\right) $$

Where:

*n* = sample size.

*d* = design effect.

*p*_1_ = the prevalence of modern contraception.

*p*_2_ = projected prevalence at the end of the study, so that (*p*_2_ − *p*_1_) is the magnitude of change you want to be able to detect.

*p =* (*p*_1_ +*p*_2_)/2.

*Z*_1 − *α*_= the z-score corresponding to desired level of significance.

*Z*_1 − *β*_ = the z-score corresponding to the desired level of power.

The table below shows the required sample size for each group with varying magnitude of change in proportions to be detected, different power sizes, and two-sided level of significance of 0.05.

The study will require a sample size of 435 participants per randomized group in order to obtain 90% power to detect a 15% change in the prevalence of modern contraception at a two-sided of 5% level of significance. The sample size estimations assumed no design effect i.e. d = 1. After adjusting for the 10% non-response rate, a sample size of 479 was computed leading to a total sample size of 958.

### Participant recruitment

We propose to randomly recruit the study participants from the university halls and private affiliated hostels at KYU. A listing of university halls and affiliated hostels and their estimated population will be prepared with the help of student leaders at the university. Permission to access the halls and hostels will be sought from the respective wardens. We will further conduct a listing of room numbers across the university halls and hostels identified. Rooms will be uniquely identified by the name of hall/ hostel and the room number. A random sample of 958 rooms will be taken and, in every room, only one participant who meets the criteria will be selected. In the event that there is more than one eligible participant in the room, a simple draw lot will be used to select one participant. Several iterations of this sample plan will be made till when the required sample size is achieved. We aim at recruiting 70% females and 30% males on the program. Trained research assistants will move to the selected rooms and recruit the study participants. Recruited participants will first be screened for eligibility before enrollment in the study.

### Randomization and allocation

Enrolled participants will be randomized to two arms of the study in a 1:1 ratio, participants that will receive the App and control participants that will not receive the App. At the time of consent and enrollment, we shall explain to the participants that there is a 50% chance that they receive the app, that the research team will have no control over this, and will not know until the end of the study whether or not they were randomized to receive the app. We will only record the telephone number of the consenting participant, link this to a unique study ID and pass this on to the app developer. The app developer will use a computer generated random sequence of numbers, matched to telephone numbers and study ID to allocate participants to the trial. The app will be available for download from the developer’s repository and will be password protected. To ensure that the treatment is not contaminated, the developer will use a unique one-time password, only useable once by a person (telephone number/ username) that have been randomized to receive the app. Participants randomized to receive the app will receive text messages with the app link and guidelines on how to install the app on their phones. They will further receive more guidelines on how the app will be used and its benefits.

### Blinding/allocation concealment

We shall make every attempt to blind the research team at GHE Consulting to the allocation sequence. Only the app developer will know or have access to this sequence. We propose to use an electronic system of data collection that is managed by the app developer. The statistician at GHE Consulting will have access only to data from the standard questionnaire to track response rates. The app developer will retain all data on app usage and downloads until the end of the study.

### Baseline and follow-up assessments

Enrolled study participants will be followed up at one and six months to determine the effectiveness of *the* SRH App in increasing SRH awareness and uptake*.* A standardized questionnaire has been developed for this study. At enrollment, the research assistant will obtain background information from eligible and consenting participants. We will collect data on the demographic and socioeconomic characteristics, SRH knowledge, sexual behavior and attitude, and SRH uptake (contraception and sexually transmitted infections testing and treatment). Information on SRH uptake will be self-reported at both baseline and end line. Questions on SRH knowledge and sexual behaviors will be modified from the World Health Organization (WHO) SRH guiding questionnaire [40] and the Uganda AIDS Indicator Survey [11]. Questions on SRH uptake will be obtained from the UDHS questionnaire. The questionnaire will be administered to consenting participants at enrollment. At the close of the study (after un-blinding the GHE (U) Ltd. team), we shall conduct an acceptability and user satisfaction survey among participants in the intervention arm of the study. We will also follow up the recruited participants in the two study arms with the standardized questionnaire similar to the baseline questionnaire to assess the impact of the SRH App on program indicators.

### Data collection procedures

We plan to use the smart-phone based Open Data Kit (ODK) app to collect data both at baseline and end line. Research assistants will be trained on the use of smart-phone and ODK for data collection. This will enable the team to collect high-quality data in real time, thereby saving time.

The app developer will also track app download and usage statistics so that at the close of the study we can determine who of those randomized to the study actually downloaded and used the app to access SRH tools and services. We believe this is not only a cost-efficient way to collect the data but will contribute to blind assessments as the GHE Consulting team will not have direct access to mobile contacts. While app use will be managed by the developer, the PI will have access to user statistics through the administrator module of the app.

### Data management

We will separate the responsibility of GHE Consulting and the app developer for data management to ensure the integrity of the research and confidentiality. All data collected at all times will not be identifiable. The app developer will maintain telephone contacts and the random allocation sequence, while the statistician at GHE will maintain app usage statistics. GHE Consulting will maintain records of the standard questionnaire responses and will administer the end-line acceptability and user satisfaction questionnaire. These two data systems will be maintained separately and only linked through unique study IDs.

The app developer will hand over all data to GHE (U) Ltd. at the close of the study. Direct access to analyze, verify and reproduce study data or reports will be granted to the PI, research coordinator, and study statistician. Regulatory entities like Grand Challenges Canada (GCC) and the MUST IRB that may want to monitor the study will be granted access to study procedures and data where requested. Pursuant to our contract with Grand Challenges Canada, the original source documents, study records, and reports will be stored intact for a period of ten years after the study ends.

### Study outcomes

The primary outcome of the study is the magnitude of change in the SRH services uptake measured by the proportion change of modern contraceptives use and testing and treatment for sexually transmitted infections at month six. The secondary outcomes are changes in participant’s knowledge of SRH and sexual behaviors at month six.

### Primary analysis

We will assess the differences at baseline between the intervention and control arms. Observed group differences for each variable will be summarized using means and proportions. The primary analysis will be an intention to treat analysis (ITA). The magnitude of change in proportion between the two groups provides a potentially unbiased estimate of the average treatment effect (ATE) of the SRH App. We will use a two-sample proportion Z-test to test the null hypothesis that the two sample changes in proportions are significantly different at month six at 5% level of significance. We will also carry out a difference in difference (DID) analysis to identify the magnitude of change as a result of the SRH App. We will also compare the changes in the different contraceptive methods and STI tests self-reported at six months by study arm.

We will conduct binary regression analysis with the primary outcome as the response variable and the participant characteristics as the explanatory variables. We shall adjust for any confounding influence that may arise from the participant belonging in any study arm or time point of data collection by adding the study arm and time point as explanatory variables in the regression model.

### Secondary analysis

We are also interested in the effect of the SRH App among those who download and use the App. Using the data from the app developer, we shall construct a binary index of app use. We shall then conduct an instrumental variables analysis to obtain an estimate of the effect of app use on SRH uptake and awareness. We shall conduct the standardized test to verify whether the instrument has good predictive power in the first stage of the regression and whether it is valid in a multiple regression analysis with the observed characteristics included in the model.

We also further assess if SRH awareness is associated with changes in SRH uptake among the study participants in both arms of the study. We will construct an index for SRH awareness and the conduct a binary regression analysis with SRH uptake as the response variable and SRH awareness as the explanatory variable. We check the validity of the association in a multiple regression analysis with observed characteristics included in the model. Both adjusted and crude odds ratios with their corresponding confidence intervals and *p*-values will be reported. All results will be considered statistically significant at a 5% level of significance.

To assess acceptability and user-satisfaction, we will analyze the download and usability statistics and reviewer comments on the app from those randomized to receive the app. Further, we have included questions on acceptability and user satisfaction (user rating of the app) and will report simple proportions for each question.

### Quality assurance of the SRH App services

We have identified three quality assurance areas for services provided using the app: 1) quality of SRH services and products provided by the clinics and pharmacies, 2) preventing contamination of the tools on delivery, and 3) ensuring that the product is received by the user that ordered for it. We propose to recruit registered and certified clinics and pharmacies to provide the SRH App services. Partner clinics and pharmacies will be recruited by GHE Consulting medical and pharmacy specialists. Product will be delivered in sealed packages. The user will receive the notification that the order has been sent and after receiving it they will confirm and the notification will be sent to the sender that the order has been received. We will also partner with SafeBoda that are identifiable, trackable and promote safety in the bodaboda business in the country. The app will include a live chat feature, allowing users to ask questions and make complaints about services delivery.

b) Cost-effectiveness and budget impact analysis

We propose to conduct a model-based incremental cost-effectiveness analysis (CEA) and budget impact analysis (BIA). The main outcomes of the economic evaluation will be the average cost per app user, cost per app service provided, and cost per disability-adjusted life year (DALY) averted. The cost per app user will be stratified distance measured in kilometers. The crude estimate of distance will be estimated by the distance between the health facility and the pickup point. We also propose to perform a static aggregate budget impact analysis (BIA) to examine the potential economic value of scaling up the App services delivery to the government and the individual taxpayer.

We will perform a primary micro-costing of the SRH App services delivery model from both the payer’s perspective, i.e. include all direct medical and all direct non-medical costs, and the modified societal perspective, i.e. add indirect costs of lost productivity as a result of app use. The direct medical costs will include all resources used in providing SRH services in terms of the medical devices and supplies. The direct non-medical costs will include transport costs incurred in delivering the services, communication costs involved, app administration costs, other program costs and the opportunity costs. Data on costs will be collected through the app use statistics and health facility personnel costs.

We will also estimate the aggregate national budget impact as well as the cost per Ugandan taxpayer per year of implementing the SRH app services delivery model. The BIA will involve the following steps: 1) characterization of the population of Uganda to estimate the potential number of people to be impacted by the app, 2) selection of a time horizon between 1 and 3 years, 3) documenting the current state of SRH services demand creation models nationally, 4) estimating the cost of SRH App services delivery model at scale, and 5) presenting budget impacts tailored to different audiences. The main analysis will be the generation of an estimate of the potential annual fiscal implication for the taxpayer and the government.

c) In-app advertising

We propose to develop the advertisement interface in the app to test a way of generating revenues to the sustain the subsidies that will be extended to the users of the SRH App. We will develop high-impact pitches and approach the different companies like telecommunication companies, hospitals, and pharmacies, beverage and breweries companies, among others and invite them to buy advertising space in the app. We will develop metrics for cost estimation and content for the different adverts based on the Uganda Communications Commission guidelines and market prices and sell advertising space to corporations.

We will assess companies’ willingness to advertise with the app at scale-up through the key informant interviews. A key informant interview is attached. We will also assess the satisfaction level of app users on the advertisement interface. Satisfaction questions are included in the acceptability and user satisfaction questionnaire.

#### Local regulatory requirements

We do not anticipate any local ICT regulatory requirements either from the Uganda Communications Commission or the Ministry of Information and Communications Technology. However, should these arise we are prepared to present all necessary documentation. We will involve the BCC experts in the Ministry of Health and the IT experts at the National Information Technology Authority (NITA) and we shall share all iterations of the app with both the Ministry, NITA and MUST IRB.

#### Ethical considerations

This study will be submitted for approval by the Mbarara University of Science and Technology (MUST) Institutional Review Board (IRB) and the Uganda National Council of Science and Technology (UNCST). All investigators have or will receive ethics training prior to the start of the study. All participants will provide informed consent and will be allowed withdraw consent at any time. Study documents will be kept under highest standards of confidentiality and records will be kept under lock and key.

This is a data-sensitive project, especially with regard to confidentiality of participants’ mobile phone contact numbers, and SRH service and tools clinical records. We will make every effort to limit access to the client records. We shall discuss possible encryption technologies with the app developer. In any case, it is only the app developer who will have access to telephone numbers, and all records of numbers will be destroyed at the completion of final data collection phase.

The SRH App poses minimal risks to the user. In the event that the user registers any adverse event or side effect as a result of the use of SRH app tools and services, they can make an appointment with our clinic or go to the nearby partner clinic for help. We will report any such cases to MUST review board within 7 days of the occurrence.

#### Study status

The study has already hired an experienced app developer, conducted the first set of Focus Group Discussions, and the app prototype is currently under development.

## Discussion

In this pilot study, we will develop a sexual and reproductive health services delivery and awareness phone application and assess its effectiveness in increasing awareness and uptake of sexual and reproductive health services among the youth aged 18 to 30 years using a randomized controlled design. The study will also assess the acceptability, usability, cost-effectiveness of this services delivery model. Further to this, the study will also assess effectiveness of in-app advertising as a way of generating revenues possible transition to scale. Existing evidence reveals that sexual and reproductive health programs tailored to the youth should be accessible, equitable, appropriate and effective [17]. The challenges affecting the youth and adolescents in low-income nations like Uganda include privacy, confidentiality, and limited SRH knowledge [11]. We hypothesize that the proposed App that will allow the youth to order for SRH tools and have them delivered to their preferred pick-up points presents an a private, confidential, youth-friendly and effective method of creating demand for sexual and reproductive health tools among the youth. The App will also enable the users to book for the services online from a pool of the partner clinics, and also have access to sexual and reproductive health information tips. The intervention provides subsidies inform of price cuts on the products and services to further incentivize the low-income youth to embrace safe sexual practices, increase use of family planning services, and other sexual and reproductive health services. Thus, our innovation reduces both the knowledge and financial barriers to access of SRH information and services by providing comprehensive SRH information and subsidizing SRH services. In the long run, the SRH App will be self-sustaining through revenues from in-app advertisements. The study helps in understanding 1) the causal effect of mobile phone applications and SRH service demand creation and uptake among youth age 18–30, 2) the cost-effectiveness of such interventions in low-income settings, and 3) the use of in-app advertisements for revenue generation to SRH services. If the study is successful, it will present policymakers and other stakeholders in Uganda, and internationally, with a viable services delivery model for increasing uptake of SRH services, tools, and awareness among the youth. The strength of this study lies in it’s innovativness to leverage mobile phones to provide the non-existent servics delivery model in the line of sexual and reproductive health services. The further applies a rigorous study design-a randomized controlled study design to evaluate the effcetiveness of the App. Even though the study is controlled, we are not able to control students in the control group interacting with with students in the trial arm of the study. This has potential for bias and a possibility of App services mis-use for tools that will be ordered online.

## Conclusion

This paper has described the steps that will be taken to develop and evaluate the impact of mobile phone application in increasing awareness and uptake of SRH services among the youth in Uganda. Unlike existing non-self-sufficient programs, this study will provide evidence on the use of in-app purchases as a means of funding the transition to scale. The pilot study will culminate into a demand-driven, culturally-relevant, and easy-to-use mobile app that will be popularly used and effective among the youth in Uganda.

## Abbreviations

ATE: Average treatment effect
BIA: Budget impact analysis
CEA: Cost effectiveness analysis
DALY: Disability adjusted life years
DID: Difference in difference
FGDs: Focus Group Discussions
GCC: Grand Challenges Canada
GHE: Global Health Economics
HIV: Human immune virus
IRB: Institutional review board
ITA: Intention to treat analysis
KYU: Kyambogo University Uganda
LMICs: Low and middle-income countries
MUST: Mbarara University of Science and Technology
NITA: National Information Technology Authority
ODK: Open data toolkit
SDGs: Sustainable Development Goals
SRH: Sexual and reproductive Health Services
STI: Sexually Transmitted infections
UDHS: Uganda Demographic and Health Survey
UNCST: Uganda National Council of Science and Technology
WHO: World Health Organization
